# Preparation of Ultra-Smooth Cu Surface for High-Quality Graphene Synthesis

**DOI:** 10.1186/s11671-018-2740-x

**Published:** 2018-10-25

**Authors:** Longlong Zhan, Yue Wang, Huicong Chang, Richard Stehle, Jie Xu, Libo Gao, Wanli Zhang, Yi Jia, Fangzhu Qing, Xuesong Li

**Affiliations:** 1 State Key Laboratory of Electronic Thin Films and Integrated Devices & School of Electronic Science and Engineering, University of Electronic Science and Technology of China, Chengdu, 610054 People’s Republic of China; 2 Qian Xuesen Laboratory of Space Technology, China Academy of Space Technology, Beijing, 100094 People’s Republic of China; 3 Mechanical Engineering Department, Sichuan University-Pittsburgh Institute, Sichuan University Jiang’an Campus, Chengdu, 610207 People’s Republic of China; 4 National Laboratory of Solid State Microstructures, School of Physics, Collaborative Innovation Center of Advanced Microstructures, Nanjing University, Nanjing, 210093 China; 5 National Engineering Research Center of Electromagnetic Radiation Control Materials, University of Electronic Science and Technology of China, Chengdu, 610054 People’s Republic of China

**Keywords:** Graphene, Cu surface, Annealing, Electro-polishing

## Abstract

As grown graphene by chemical vapor deposition typically degrades greatly due to the presence of grain boundaries, which limit graphene’s excellent properties and integration into advanced applications. It has been demonstrated that there is a strong correlation between substrate morphology and graphene domain density. Here, we investigate how thermal annealing and electro-polishing affects the morphology of Cu foils. Ultra-smooth Cu surfaces can be achieved and maintained at elevated temperatures by electro-polishing after a pre-annealing treatment. This technique has shown to be more effective than just electro-polishing the Cu substrate without pre-annealing. This may be due to the remaining dislocations and point defects within the Cu bulk material moving to the surface when the Cu is heated. Likewise, a pre-annealing step may release them. Graphene grown on annealed electro-polished Cu substrates show a better quality in terms of lower domain density and higher layer uniformity than those grown on Cu substrates with only annealing or only electro-polishing treatment.

## Background

As a two-dimensional monolayer of sp^2^-hybridized carbon atoms arranged in a honeycomb lattice, graphene has recently had a strong focus in academia and in industry due to its extraordinary properties [1–4]. Chemical vapor deposition (CVD) [5] growth of graphene on metal catalytic substrates, e.g., Cu, has been shown to be the most promising method to date for the growth of large-area and high-quality graphene films [6]. However, degraded greatly by grain boundaries [7–9], CVD-grown graphene films are typically polycrystalline [10], limiting its integration into advanced technological applications. Therefore, synthesizing graphene with minimal crystalline defects and low domain density by eliminating the negative effects of grain boundaries is of great importance [11].

It has been demonstrated that there is a close correlation between substrate morphology and graphene nucleation sites [12–14]. CVD growth of graphene is typically performed on commercial polycrystalline Cu foils. As-received Cu prepared by a cold rolling process often has many defects [12, 15, 16], such as rolling lines, potential strains, impurities, and native oxide, which greatly impact the quality of the graphene. To improve the morphology of copper, a wide variety of pretreatment methods have been investigated, such as annealing [17–24], physical polishing [25], etching [15, 26], electro-polishing [13, 27–30], liquefying [31], and melting-resolidification [32]. Among them, annealing and electro-polishing are the most widely employed due to increased efficiency and convenience. With the rearranging of Cu surface atoms, releasing internal stress in copper and growing Cu crystal size, annealing has become an indispensable step in graphene growth [21–23]. However, limited by the formation of step bunching and evaporation of Cu atoms [23, 33], the surface of annealed Cu remains relatively rough which has a negative influence on graphene growth. Electro-polishing treatments can significantly improve the surface morphology of the substrate, which is critical to obtain homogenous graphene films as well as avoiding graphene adlayer formation [27, 34]. However, the defects of Cu such as etching pits and spike points are still hard to avoid by traditional electro-polishing techniques [28, 29]. Therefore, techniques to prepare ultra-smooth metallic substrates need to be investigated and improved upon.

In this work, we combined annealing and electro-polishing together for the preparation of smooth Cu substrates. Although electro-polishing is an efficient method to make smooth surfaces, graphene growth is normally conducted at high temperatures which may release the internal strain and move dislocations to the surface. This could cause the Cu surface to be roughened again. Here, we annealed the Cu substrate before electro-polishing to release the residue strain and defects. In this way, the surface reconstruction due to strain release when growing graphene at high temperatures was significantly restricted and the electro-polished surface could be maintained. We demonstrated that the domain density of graphene grown on such Cu substrates is greatly reduced compared to those on just an annealed or an electro-polished Cu substrate. Our method to prepare smooth substrates benefits the synthesis of not only graphene but also other thin-film or two-dimensional materials.

## Methods

### Cu Foil Preparation

For *as-received Cu* (AR-Cu), Cu foils are from Alfa Aesar (25 μm, 99.8%, #46365).

For *annealed Cu* (AN-Cu), the AR-Cu foils were annealed at 1050 °C in hydrogen under 6.8 Pa for 1 h.

For *electro-polished Cu* (EP-Cu), the test Cu foil is used as the anode and a second piece of satisfying Cu foil as the cathode. The electrolyte consists of 500 ml phosphoric acid, 250 ml acetic acid, and 250 ml isopropyl alcohol. The current density is about 47 A/m^2^. The polishing time is 30 min.

For *electro-polished annealed copper* (EA-Cu), the Cu foil is annealed and then electro-polished.

For *annealed electro-polished copper* (AE-Cu), the Cu foil is electro-polished and then annealed.

### Graphene Growth and Transfer

In this work, a common atmospheric pressure CVD system was used to grow graphene, equipped with a dry mechanical vacuum pump [35] (Chengdu Hao-Shi Technology Ltd.). For graphene growth, various Cu substrates (2 × 1 cm^2^, respectively) were put on a quartz plate and heated to 1050 °C at a rate of 17.5 °C/min. Then, the substrates were annealed at atmospheric pressure with 200 sccm argon (Ar) and 4 sccm H_2_ flow at 1050 °C for 30 min. After annealing, 1 sccm flow of 1% CH_4_/Ar mixture was introduced to the chamber for graphene growth. Isolated domains or continuous films were achieved by controlling the growth time. The Cu foils were placed in parallel so as to exclude the effect led by the difference of the gas transportation [36].

Graphene transfer was conducted with the PMMA-wet transfer method [5]. Two hundred eighty-five-nm-thick SiO_2_/Si wafers were used as the support substrates.

### Characterization

Optical microscopy (Nikon, ECLIPSE LV100D), atomic force microscopy (AFM; Veeco D5000), Raman spectroscopy (Renishaw Invia, *λ* = 532 nm), and van der Pauw-Hall measurements (VDP-H; Copia, HMS-5000) were conducted for detailed characterizations. For van der Pauw-Hall, about 1 × 1 cm^2^ transferred graphene samples were annealed in the CVD chamber under vacuum at 200 °C to remove the adsorbed gas in air first and then characterized.

## Results and Discussion

### Cu Foil Preparation

**Fig. 1:**
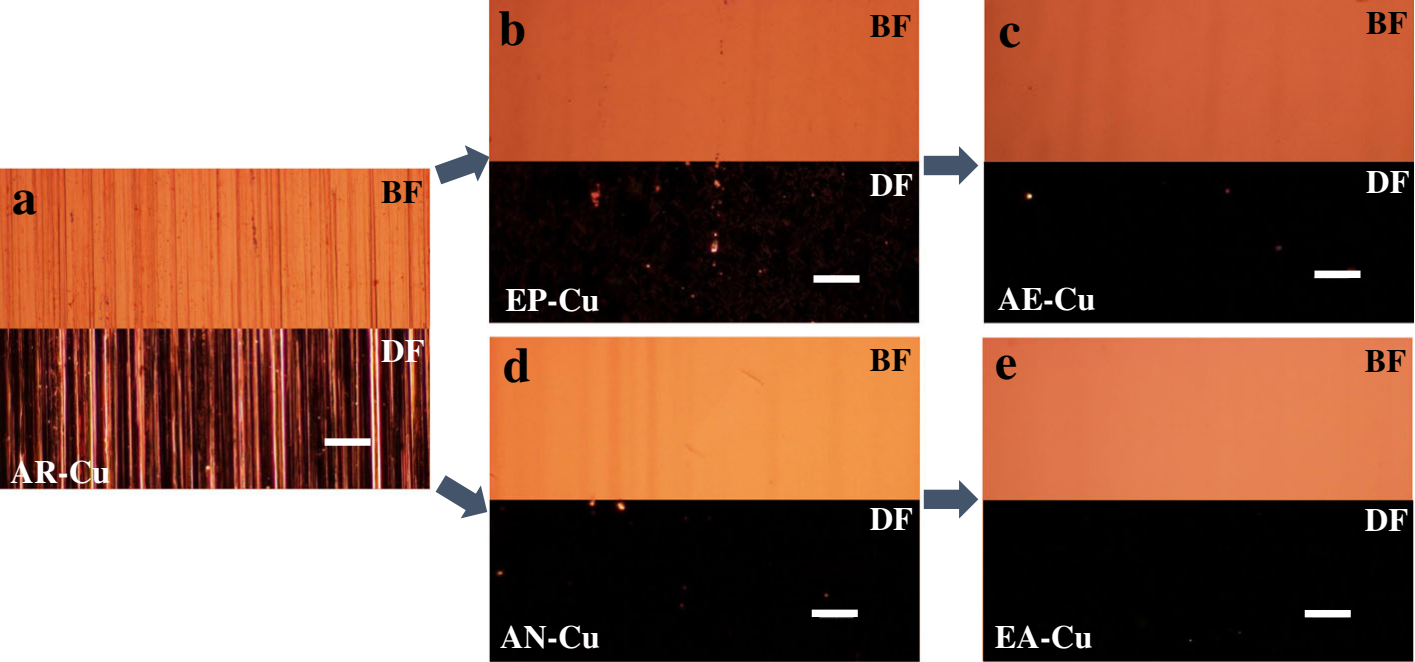
OM images of Cu foils with different pre-treatments under bright and dark fields. **a** AR-Cu, **b** EP-Cu, **c** AE-Cu, **d** AN-Cu, and **e** EA-Cu, respectively. Scale bars, 20 μm

Figure 1 shows the morphologies of the Cu foils prepared with different treatments by optical microscopy (OM). As shown in Fig. 1a, the surface of AR-Cu displays large corrugation in both bright field (BF) and dark field (DF). From Fig. 1b–e, it can be seen that the pretreated Cu substrates have smoother surfaces.

**Fig. 2:**
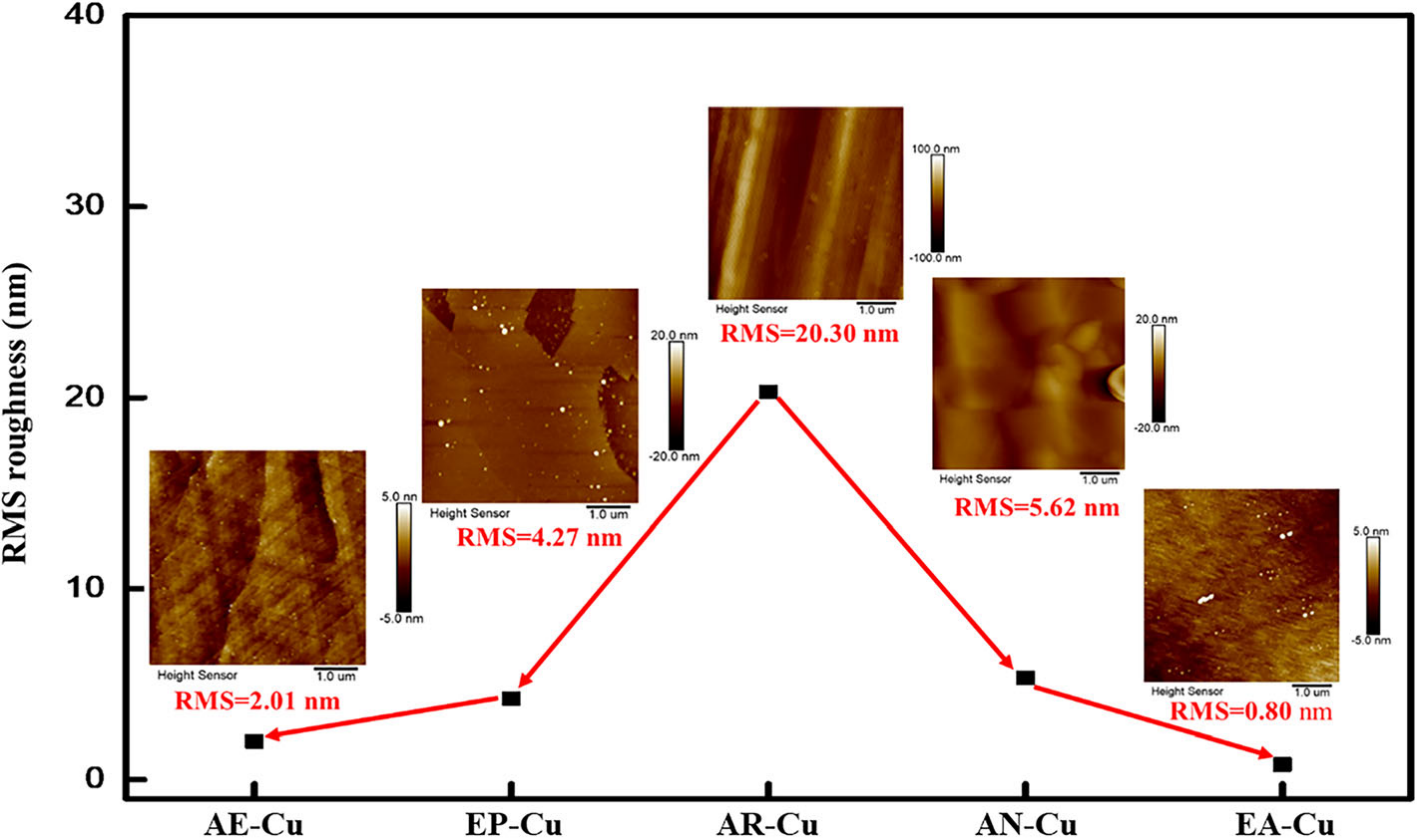
Average RMS roughness evolution (black squares) of the Cu surface after each processing step obtained in AFM

Atomic force microscopy (AFM) characterization provides quantitative understanding on different treatment methods, as shown in Fig. 2. Apparently, the AR-Cu has a really rough surface with the root mean square (RMS) roughness of 20.30 nm. As reported, both thermal annealing and electro-polishing can effectively smoothen the surface [12, 18, 27, 37], reducing the surface roughness to 5.62 nm and 4.27 nm, respectively. In addition, a combination of thermal annealing and electro-polishing, i.e., either thermal annealing after electro-polishing or electro-polishing after thermal annealing, can further reduce the surface roughness to 2.01 nm and 0.80 nm, respectively. The surface of the EA-Cu being smoother than the AE-Cu can be attributed to the fact that thermal annealing can help to release the residue internal strain and dislocations. Thus, if the Cu substrate is electro-polished after annealing, as the residue internal strain and dislocations have been released, the surface can be well polished. On the other hand, if the Cu substrate is annealed after electro-polishing, although a smooth surface can be achieved by electro-polishing, during the annealing process, the surface may be reconstructed due to the release of the internal strain and the motion of the dislocations to the surface and thus the final roughness is impacted.

### Graphene Growth

**Fig. 3:**
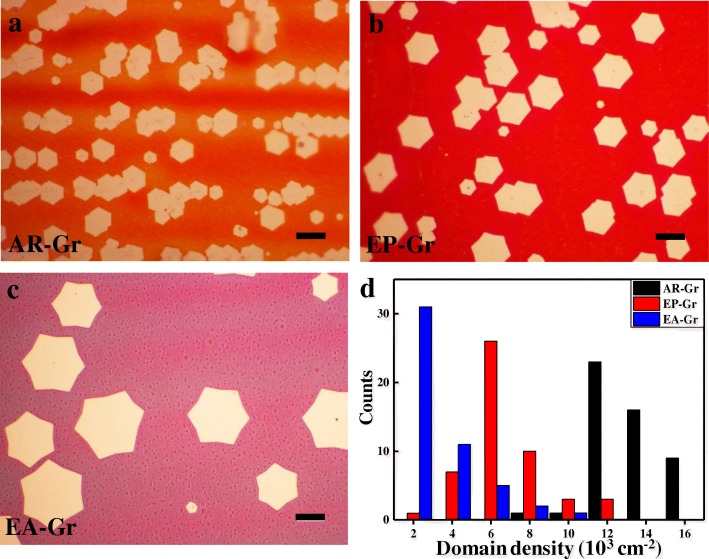
OM images of graphene domains grown on **a** AR-Cu, **b** EP-Cu, and **c** EA-Cu, respectively. Scale bars, 10 μm. **d** Histogram statistical graph of graphene domain density on AR-Cu, EP-Cu, and EA-Cu, respectively. The domain density is calculated by randomly taken a region with an area of 120 × 90 μm^2^ and then counting the domains within the region

It has been reported that graphene domain density and thickness uniformity are correlated to the surface roughness of the Cu substrate [12, 23, 34, 38]. From Fig. 3a–c, it can be seen clearly that the graphene domain density decreases with the decrease of the Cu surface roughness. The domain density of graphene on AR-Cu (defined as AR-Gr) is considerably high up to 1.16 × 10^4^ cm^−2^ (Fig. 3a). That of graphene on EP-Cu (defined as EP-Gr) drops by 2.25 times, with only 5.2 × 10^3^ cm^−2^ (Fig. 3b). That of graphene on EA-Cu (defined as EA-Gr) further drops to 1.7 × 10^3^ cm^−2^, 7.3 times lower than that of AR-Gr and 3.2 times lower than that of EP-Gr (Fig. 3c). Figure 3d shows the statistical analysis of the graphene domain density on the three surfaces (AR-Cu, EP Cu, and EA-Cu, respectively), which quantitatively show the effect of Cu surface roughness on graphene nucleation density. All are consistent with previous work. It can also be seen that the growth rate of EA-Gr is greatly enhanced compared to the other two Cu foils.

**Fig. 4:**
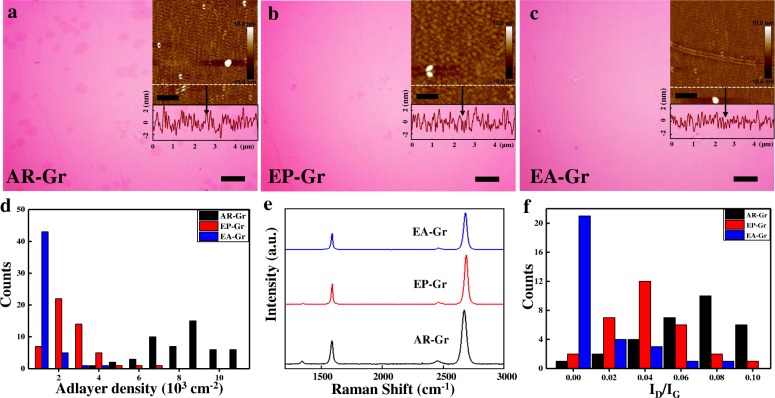
OM images of transferred graphene films grown on **a** AR-Cu, **b** EP-Cu, and **c** EA-Cu. Scale bars, 10 μm. (AFM images and amplitude spectrum corresponding to each transferred graphene, inset upper right corner. Scale bars, 1 μm.) **d** Histogram statistical graph of graphene adlayer density grown on AR-Cu, EP-Cu, and EA-Cu. The adlayer density is calculated by randomly taking a region with an area of 120 × 90 μm^2^ and then counting the adlayers within the region. **e** Raman spectra of transferred graphene grown on AR-Cu, EP-Cu, and EA-Cu, respectively. **f** Histogram statistical graph of *I*_D_/*I*_G_ in Raman spectra of graphene grown on AR-Cu, EP-Cu, and EA-Cu

The OM images of the transferred graphene with typical distribution of adlayers are shown in Fig. 4a–c, and the histogram statistical graph of graphene adlayer density is shown in Fig. 4d for AR-Gr, EP-Gr, and EA-Gr, respectively. As expected, the smoother the surface, the less adlayers. The AR-Gr is inhomogeneous with many adlayers, with an average adlayer density of 7.3 × 10^3^ cm^−2^ (Fig. 4a). The adlayer density of EP-Gr is reduced by four times with only 1.8 × 10^3^ cm^−2^(Fig. 4b). The EA-Gr is the most homogeneous with the adlayer density only about 2 × 10^2^ cm^−2^, 36 times lower than that of AR-Gr and 9 times lower than that of EP-Gr. AFM images corresponding to each transferred graphene are also shown, inset upper right corner. The spectral RMS amplitude of AR-Gr, EP-Gr, and EA-Gr are 245.2 pm, 175.7 pm, and 94.2 pm, respectively. The transferred EA-Gr shows the smoothest surface morphology.

One of the major reasons to reduce graphene domain density is that the domain boundaries are thought to be one of the defects deteriorating graphene quality, e.g., electrical transport performance. Raman spectroscopy is commonly used for graphene characterization and the intensity ratio of the D band to the G band (*I*_D_/*I*_G_) is correlated to graphene defect density [39]. Figure 4e, f shows the Raman spectra and histogram statistical graph of ID/IG of the three kinds of graphene. The EA-Gr has the most perfect crystalline structure with nearly no D peak. Generally, *I*_D_/*I*_G_ is ~ 10 ± 5% for the AR-Gr, ~ 5 ± 2% for EP-Gr, and ~ 1 ± 1% for EA-Gr. That is, the smoother the substrate surface, the higher the quality of graphene.

### Electrical Transport Performance of Graphene

**Fig. 5:**
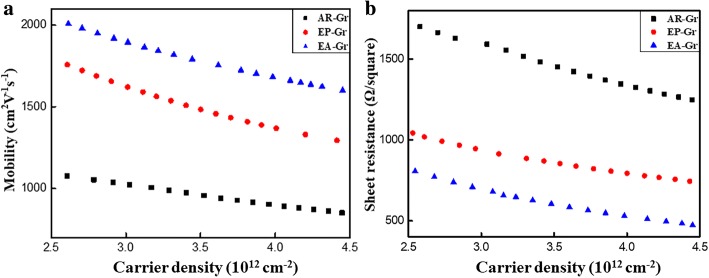
Plot of graphene **a** carrier mobility vs carrier density and **b** sheet resistance vs carrier density at room temperature

The van der Pauw-Hall measurement is commonly used to characterize the electrical transport performance of thin films. Sheet resistance, carrier density, and carrier mobility can be measured or derived. However, in most of the cases, the measured carrier mobility from different graphene samples do not correspond to the same carrier density due to the unintentional doping from the surroundings. For these cases, the carrier mobility is not comparable because it is a function of carrier density [40, 41]. Here, we conducted the van der Pauw-Hall measurement on annealed graphene, which had an initially low carrier density. The carrier density increased with time due to the dopant adsorption from the surroundings and the corresponding carrier mobility could be measured. The measured carrier mobility and sheet resistance as a function of carrier density for the three kinds of graphene are shown in Fig. 5. It can be seen that the EA-Gr shows the best transport performance with the highest carrier mobility and the lowest sheet resistance.

## Conclusions

In summary, we presented an efficient route to prepare ultra-smooth substrates by first annealing and then electro-polishing commercial copper, which is more effective in achieving a smooth surface than just annealing or electro-polishing alone. This is attributed to the fact that thermal annealing can release the residue internal strain and dislocation, thus the smooth surface achieved by electro-polishing can be preserved at elevated temperatures for graphene growth. The efficiency of the smooth surface prepared in this way was demonstrated by the reduction of graphene domain density, adlayer density, defect density, and the improvement of electrical transport performance.

## Abbreviations

AE-Cu: Annealed electro-polished Cu
AFM: Atomic force microscopy
AN-Cu: Annealed Cu
AR-Cu: As-received Cu
AR-Gr: Graphene grown on AR-Cu
BF: Bright field
CVD: Chemical vapor deposition
DF: Dark field
EA-Cu: Electro-polished annealed Cu
EA-Gr: Graphene grown on EA-Cu
EP-Cu: Electro-polished Cu
EP-Gr: Graphene grown on EP-Cu
OM: Optical microscopy
RMS: Root mean square

## References

[1] Kim HH, Chung Y, Lee E (2014). Water-free transfer method for CVD-grown graphene and its application to flexible air-stable graphene transistors. Adv Mater.

[2] Lee C, Wei X, Kysar JW (2008). Measurement of the elastic properties and intrinsic strength of monolayer graphene. Science.

[3] Nair RR, Blake P, Grigorenko AN (2008). Fine structure constant defines visual transparency of graphene. Science.

[4] Zhang Y, Tan Y-W, Stormer HL (2005). Experimental observation of the quantum Hall effect and Berry’s phase in graphene. Nature.

[5] Li X, Cai W, An J (2009). Large-area synthesis of high-quality and uniform graphene films on copper foils. Science.

[6] Li X, Colombo L, Ruoff RS (2016). Synthesis of graphene films on copper foils by chemical vapor deposition. Adv Mater.

[7] Huang PY, Ruiz-Vargas CS, van der Zande AM (2011). Grains and grain boundaries in single-layer graphene atomic patchwork quilts. Nature.

[8] Yazyev OV, Louie SG (2010). Electronic transport in polycrystalline graphene. Nat Mater.

[9] Yu Q, Jauregui LA, Wu W (2011). Control and characterization of individual grains and grain boundaries in graphene grown by chemical vapour deposition. Nat Mater.

[10] Li X, Cai W, Colombo L (2009). Evolution of graphene growth on Ni and Cu by carbon isotope labeling. Nano Lett.

[11] Qing F, Shen C, Jia R (2017). Catalytic substrates for graphene growth. MRS Bull.

[12] Han GH, Guenes F, Bae JJ (2011). Influence of copper morphology in forming nucleation seeds for graphene growth. Nano Lett.

[13] Luo ZT, Lu Y, Singer DW (2011). Effect of substrate roughness and feedstock concentration on growth of wafer-scale graphene at atmospheric pressure. Chem Mater.

[14] Wofford JM, Nie S, McCarty KF (2010). Graphene islands on cu foils: the interplay between shape, orientation, and defects. Nano Lett.

[15] Kim Soo Min, Hsu Allen, Lee Yi-Hsien, Dresselhaus Mildred, Palacios Tomás, Kim Ki Kang, Kong Jing (2013). The effect of copper pre-cleaning on graphene synthesis. Nanotechnology.

[16] Wood JD, Schmucker SW, Lyons AS (2011). Effects of polycrystalline cu substrate on graphene growth by chemical vapor deposition. Nano Lett.

[17] Ago H, Ogawa Y, Tsuji M (2012). Catalytic growth of graphene: toward large-area single-crystalline graphene. J Phys Chem Lett.

[18] Huet B, Raskin J-P (2018). Role of Cu foil in-situ annealing in controlling the size and thickness of CVD graphene domains. Carbon.

[19] Jo Insu, Park Subeom, Kim Dongjin, Moon Jin San, Park Won Bae, Kim Tae Hyeong, Kang Jin Hyoun, Lee Wonbae, Kim Youngsoo, Lee Dong Nyung, Cho Sung-Pyo, Choi Hyunchul, Kang Inbyeong, Park Jong Hyun, Lee Jeong Soo, Hong Byung Hee (2018). Tension-controlled single-crystallization of copper foils for roll-to-roll synthesis of high-quality graphene films. 2D Materials.

[20] Kim Youngwoo, Moyen Eric, Yi Hemian, Avila José, Chen Chaoyu, Asensio Maria C, Lee Young Hee, Pribat Didier (2018). Synthesis of high quality graphene on capped (1 1 1) Cu thin films obtained by high temperature secondary grain growth on c-plane sapphire substrates. 2D Materials.

[21] Li X, Magnuson CW, Venugopal A (2010). Graphene films with large domain size by a two-step chemical vapor deposition process. Nano Lett.

[22] Mun JH, Cho BJ (2013). Synthesis of monolayer graphene having a negligible amount of wrinkles by stress relaxation. Nano Lett.

[23] Sarajlic OI, Mani RG (2013). Mesoscale scanning electron and tunneling microscopy study of the surface morphology of thermally annealed copper foils for graphene growth. Chem Mater.

[24] Xu X, Zhang Z, Dong J (2017). Ultrafast epitaxial growth of metre-sized single-crystal graphene on industrial Cu foil. Sci Bull.

[25] Dhingra S, Hsu J-F, Vlassiouk I (2014). Chemical vapor deposition of graphene on large-domain ultra-flat copper. Carbon.

[26] Murdock AT, van Engers CD, Britton J (2017). Targeted removal of copper foil surface impurities for improved synthesis of CVD graphene. Carbon.

[27] Griep MH, Sandoz-Rosado E, Tumlin TM (2016). Enhanced graphene mechanical properties through ultrasmooth copper growth substrates. Nano Lett.

[28] Roy SS, Jacobberger RM, Wan C (2016). Controlling the density of pinhole defects in monolayer graphene synthesized via chemical vapor deposition on copper. Carbon.

[29] Sridhara K, Feigelson BN, Wollmershauser JA (2017). Electrochemically prepared polycrystalline copper surface for the growth of hexagonal boron nitride. Cryst Growth Des.

[30] Tay RY, Griep MH, Mallick G (2014). Growth of large single-crystalline two-dimensional boron nitride hexagons on electropolished copper. Nano Lett.

[31] Geng D, Wu B, Guo Y (2012). Uniform hexagonal graphene flakes and films grown on liquid copper surface. Proc Natl Acad Sci U S A.

[32] Mohsin A, Liu L, Liu P (2013). Synthesis of millimeter-size hexagon-shaped graphene single crystals on resolidified copper. ACS Nano.

[33] Liu Z, Gong Y, Zhou W et al (2013) Ultrathin high-temperature oxidation-resistant coatings of hexagonal boron nitride. Nat Commun 4:2541 10.1038/ncomms3541 10.1038/ncomms3541 24092019

[34] Li Q, Chou H, Zhong J-H (2013). Growth of adlayer graphene on Cu studied by carbon isotope labeling. Nano Lett.

[35] Qing Fangzhu, Jia Ruitao, Li Bao-Wen, Liu Chunlin, Li Congzhou, Peng Bo, Deng Longjiang, Zhang Wanli, Li Yanrong, Ruoff  Rodney S, Li Xuesong (2017). Graphene growth with ‘no’ feedstock. 2D Materials.

[36] Li Z, Zhang W, Fan X (2012). Graphene thickness control via gas-phase dynamics in chemical vapor deposition. J Phys Chem C.

[37] Gnanaprakasa TJ, Gu YX, Eddy SK (2015). The role of copper pretreatment on the morphology of graphene grown by chemical vapor deposition. Microelectron Eng.

[38] Chuang M-C, Woon W-Y (2016). Nucleation and growth dynamics of graphene on oxygen exposed copper substrate. Carbon.

[39] Pimenta MA, Dresselhaus G, Dresselhaus MS (2007). Studying disorder in graphite-based systems by Raman spectroscopy. Phys Chem Chem Phys.

[40] Liu H, Liu Y, Zhu D (2011). Chemical doping of graphene. J Mater Chem.

[41] Shen C, Jia Y, Yan X (2018). Effects of Cu contamination on system reliability for graphene synthesis by chemical vapor deposition method. Carbon.

